# The history of state preemption and medical device regulation: lessons for artificial intelligence oversight

**DOI:** 10.1093/haschl/qxag046

**Published:** 2026-03-03

**Authors:** Case Thomason, Larry Bucshon, Brian J Miller, Brian Yagi

**Affiliations:** University of Michigan Law School, Ann Arbor, MI 48109, United States; Holland & Knight L.L.P., Washington, DC 20006, United States; Division of Hospital Medicine, Department of Medicine, Johns Hopkins University School of Medicine, Baltimore, MD 21205, United States; Hoover Institution, Stanford, CA 94305, United States; Division of Hospital Medicine, Department of Medicine, University of Michigan Medical School, Ann Arbor, MI 48109, United States

**Keywords:** artificial intelligence regulation, preemption, medical device regulation, Food and Drug Administration, federalism

## Abstract

Rapid expansion of artificial intelligence (AI) in health care has outpaced the United States' fragmented regulatory structure, which currently relies on overlapping albeit incomplete federal oversight from multiple agencies. In the absence of clear federal product standards, some states have advanced broad and inconsistent regulatory schemes that often sweep AI-enabled medical devices into consumer-protection frameworks not designed for clinically used technologies. These state-level requirements risk recreating the fragmented medical device regulatory landscape that existed prior to 1976, where inconsistent rules increased compliance burdens and undermined patient safety. Congress responded with the 1976 Medical Device Amendments (MDA), granting FDA jurisdiction over medical devices and simultaneously adding a preemption provision prohibiting states from enacting or enforcing requirements “different from, or in addition to” federal requirements while still respecting states' rights to regulate use in medical practice. Drawing on historical parallels to the MDA and emerging state laws, this article proposes a preemption clause limited to health care-related AI that is coupled with a modernized, flexible federal product oversight framework—grounded in the FDA's technical expertise and informed by least-burdensome principles. Patients, physicians, and entrepreneurs would gain regulatory clarity, reduced compliance fragmentation, and a supportive regulatory environment for pragmatic, responsible technological advancement.

The use of artificial intelligence (AI) in health care applications is currently subject to a piecemeal regulatory scheme involving several federal agencies, with the US Food and Drug Administration (FDA)^1^ and Assistant Secretary for Technology Policy (previously the Office of National Coordinator for Health Information Technology)^2^ leading the foray into product regulation; and the FDA,^3^ Federal Trade Commission,^4^ and Office of Civil Rights^4^ overseeing data and privacy protections. There is no unifying set of standards to ensure both consumer protection and proactively guide industry, nor for post-market surveillance of applications once deployed. For AI-enabled medical devices, clinician users and developers who want to improve efficiency, access, and safety face a lack of regulatory clarity in market entry standards that inhibits the development of safe and effective automated tools. Critically, patients and clinicians may unknowingly face unfavorable risk/benefit profiles, putting patients at risk of harm without a clear mechanism for quality or safety monitoring.

Without clear fit-for-purpose, flexible federal regulatory pathways for health care applications of AI that clearly address premarket evidentiary burden, post-market oversight, and the myriad of liability, privacy, and data security issues, many states have either decided not to regulate whatsoever, or done so by proposing and passing laws that regulate AI broadly at the state level—inclusive of health care applications. By 2024, approximately 450 AI-related bills were introduced across 45 states and the District of Columbia, with 113 of those bills enacted into law.^4^ These state proposals continue to proliferate, with approximately 260 bills introduced and 22 passed into law in the first half of 2025 alone.^5^

In the setting of the proliferation of these state laws, the 2025 One Big Beautiful Bill Act (OBBA) initially contained a broad and restrictive preemption provision^6^ (section 43201(c) of the House version) that would have barred states and localities from regulating any aspect of AI for 10 years.^7^ In the context of health care, consumer-facing tradeoffs of a 50-state vs federal product regulatory framework were not explicitly considered in the OBBA. Unsurprisingly, critics from both sides of the political spectrum viewed the provision as an overreach that nullified states' rights and their ability to protect consumers, guard privacy, or address AI harms.^8^ In the Senate, the provision was overwhelmingly struck—by a 99-1 vote^9^—with the final bill passed into law that left state and local laws intact.^10^

Currently, there are few federal guidelines for AI regulation, with a federal law banning deepfakes in specific instances.^11^ Other recent actions include the Trump Administration's White House AI Action Plan,^12^ which encourages a light regulatory touch in order to enable innovation and competition with China^13^ and promote American global technological dominance. This marks a sharp departure from the prior Biden Administration's focus on AI safety.^14^ A problem emerges where each new president may dramatically shift enforcement priorities, resulting in regulatory whiplash that undermines the pursuit of clear standards for safety and efficacy that would ultimately help patients, physicians, and hospitals through the development and deployment of pragmatic automation. In light of a changing regulatory environment, this article reviews recent state efforts to regulate AI in the absence of clear federal guardrails, the historical example of medical device regulation and associated federal preemption, and challenges with the current regulatory structure, along with future directions for policymakers.

## States' current attempts to regulate AI

As there is no federal legislation on AI regulation with a clear path to passage into law in most areas, including healthcare, the few states that have done so have instead created a patchwork of laws with broad applications.^15^ Most proposed and passed state laws contain expansive language to define AI technologies subject to state regulatory authority, and lack carve-outs or special provisions for the oversight of health care-related applications and medical devices that integrate AI-enabled software. For example, five states have targeted “high risk” AI applications as those with the “potential to significantly affect civil rights, implicate discrimination, privacy, access to critical services, health, or well-being.”^16-18^ Several states have also taken aim at AI that perform “profiling” functions, defined loosely as “automated decision making that have legal or other significant effects or a heightened risk of harm.”^17,19,20^ These passed and proposed state laws would require AI developers to produce state-level impact assessments, implement state-specific privacy/security measures, and comply with additional reporting requirements to both individual end-users and state governments. Many require the deployer (ie, health care institution or provider) of “high risk AI” to allow each individual person (ie, patient) the opportunity to opt-out of having the AI tool used in their case.^17,20^

While most of these laws have been passed under the auspices of general consumer protection, the broad language used to define the regulated AI technologies would encompass AI-enabled medical devices—making these laws a potential basis for state regulation of the safety and efficacy of medical devices. Furthermore, these laws vary in their weight of both the precautionary principle and assessment of the safety and quality performance of current models of human-driven care delivery. Emerging evidence shows that AI has potential positive impacts on care delivery, for example, in medical image interpretation by reducing human error rates^21^ and increasing diagnostic efficiency.^22^ It also now can detect melanomas from images with 100% sensitivity,^23^ automatically analyze brain CT scans in real time,^24^ and improve performance by more accurately reading CT scans,^25^ mammography,^26^ and pathology slides^27^ thus both automating components of clinical practice and enhancing the accuracy of clinical care.

While it's important to note that there are potential benefits to having state-based approaches to AI regulation,^15^ it is worth examining a historical scenario where similar consumer protection, legal, and political problems existed just prior to the enactment of the 1976 MDA,^28,29^ which introduced the federal preemption provision that provided significant benefits in the federal regulation of medical devices in the latter part of the twentieth century.^30^

## Historical background of the MDA and the necessity for federal preemption

A pivotal piece of legislation, the MDA established the framework for the FDA's regulation of medical devices. Prior to this legislation, medical devices were largely unregulated at the federal level, resulting in a patchwork of inconsistent or nonexistent oversight at the state level.^31^ The MDA introduced a long-overdue federal regulatory framework aimed at providing, at the time of its passage, what policymakers and analysts judged as a reasonable assurance of the safety and effectiveness of medical devices, while also establishing a preemption provision that limited the ability of states to impose conflicting requirements (The bill preempts State and local requirements for medical devices that differ from requirements established by the Secretary, although the Secretary may exempt a requirement of a State or locality from the preemption provision if the requirement is more stringent than the Federal requirement or if the requirement is required by compelling local conditions and if a device which complies with the requirement will not be in violation of the Act.).^31^ Through the Supremacy Clause of the US Constitution,^32^ Congress solidified the FDA as the cornerstone entity to regulate the entry of medical devices into the market and set product market standards. This federal preemption was critical to resolving the fragmented regulatory landscape that had emerged in the absence of a unified national framework.^33^

Before 1976, medical devices were not subject to meaningful, operational federal oversight. Under the original 1938 Federal Food, Drug, and Cosmetic (FD&C) Act,^34^ medical devices were technically recognized but were grouped together with drugs with respect to FDA jurisdiction.^35^ There were no premarket authorization requirements, resulting in the FDA's authority functioning as reactive—devices could be removed from the market only if found to be adulterated (eg, made under unsanitary conditions) or misbranded (eg, false labeling). Moreover, the FDA faced significant difficulty in getting courts to uphold their enforcement actions, as courts struggled to fit medical devices into the legal definition of drugs—often leading to legal rulings that the device at issue was outside the FDA's authority.^36^ Patient safety was compromised in the absence of proactive, premarket mechanisms to prevent unsafe or ineffective devices from entering the market in the first place.

This left the door wide open for a proliferation of dangerous devices or fraudulent medical claims regarding therapeutic value.^37^ For instance, quack technologies such as Ruth B. Drown's “Radio Therapeutic Instrument” gained popularity in the 1940s and 1950s despite having no scientific basis.^38^ Marketed as a cure for everything from minor ailments to terminal illnesses,^39^ Drown's device offered a false sense of hope—and in many cases, contributed to tragic outcomes.^39^ In one court case, a woman died of breast cancer that eventually progressed to widespread carcinomatosis after using the machine for years under the mistaken belief that it was effectively treating her disease.^38^ Some experts have expressed concern that AI presents a similar challenge today for regulators.^40^

Rather than requiring premarket approval, the FDA was forced to pursue civil law enforcement actions after patient harm had already occurred. The agency had to file separate cases in different states, litigating whether a product qualified as a “drug” under federal law. If so, this would enable the agency to participate in lawsuits and proceed through the trial process,^41^ a lengthy, resource-intensive, and impractical process.^42^ Additionally, the tort liability system available to victims through private lawsuits was also insufficient, as it only provided options after injuries occurred and was often slow, uncertain, and expensive. Many companies went bankrupt due to the cost of prolonged litigation, leaving victims without meaningful compensation or recovery.^41^

The inadequacy of federal oversight during this period created a regulatory void,^43^ prompting individual states—especially California, which statutorily required premarket approval of all new medical devices by the state and compliance with good manufacturing practices, including inspections^31^—to step in and enact their own medical device regulatory frameworks through state law. While these state-level actions were well-intentioned, they also resulted in regulatory fragmentation as both clinician-entrepreneurs and device manufacturers were forced to navigate a confusing array of requirements, often facing conflicting demands across state jurisdictions. This lack of uniformity both raised entry costs in some states while simultaneously providing a clear lane for bad actors to enter other geographic markets whose laws allowed for lower product market standards.

In the early 1960s, federal efforts to reform device regulation gained momentum. President John Kennedy advocated for stricter controls on medical devices,^44^ and Congress held hearings on the subject (Senate Judiciary Committee; Antitrust and Monopoly Subcommittee).^45^ These efforts, however, lost steam after the thalidomide tragedy in Europe shifted the legislative focus back to pharmaceutical product regulation. A companion bill to the 1962 drug amendments^46^ that would have required premarket approval for medical devices was ultimately dropped as Congress instead focused on the monumental step of requiring drugs to demonstrate safety and effectiveness prior to marketing approval.^47^

Meaningful changes to the medical device regulatory framework regained attention in the 1960s. Amid the increasing sophistication of medical technology—such as prosthetic joints in the early 1950s^48^ and implantable devices like pacemakers in 1960^49^—the risks associated with inadequate oversight became more apparent. For example, the growing use of pacemakers led to several lawsuits when the devices either failed to fire, fired inappropriately, or experienced hardware failure.^50,51^ Another prominently harmful device was the Dalkon Shield, a birth control device that caused serious health complications, including pelvic inflammatory disease, sterility, septic abortions, and death.^52-55^ Over 200 000 claims were filed regarding injuries by women who had used the device, leading the company to go bankrupt, while most victims were only given small awards ranging from approximately $750 to $2000.^56^

The public response was overwhelming, and with increasing injuries from medical devices that had received no premarket review, along with the rising number of cases the FDA had to handle nationwide, the federal government began to take action.^31^ In 1969, President Richard Nixon decided to endorse a full-scale examination of medical device safety.^57^ The resulting Cooper Committee, named after its chairman, Dr. Theodore Cooper of the National Institutes of Health, was tasked by the Secretary of Health, Education, and Welfare with evaluating the existing system and recommending reforms.^37^

The Cooper Committee's findings were pivotal, finding that devices were responsible for 751 deaths and over 10 000 injuries in a 10-year span.^31^ It recognized that devices were fundamentally different from drugs in their design, manufacture, and clinical use. It thus recommended a specifically tailored regulatory approach that remains the cornerstone of device regulation today: a risk-based classification system and the creation of distinct pathways for evaluating new and existing devices based on their risk profile.^58^ Importantly, it highlighted the urgent need for national legislation to ensure consistency and prevent states from implementing conflicting regulations that could undermine public health or hinder innovation.

The FDA, frustrated by Congressional inaction, took the unusual step of implementing the Cooper Committee's recommendations on its own initiative.^37^ The agency conducted an inventory of medical devices on the market, identifying approximately 8000 devices from 1100 different manufacturers, and began classifying them according to their potential risks.^43^ This assertive move finally galvanized Congress into action, and by 1976, a bipartisan consensus had formed around the need for comprehensive reform. In 1976, President Gerald Ford signed the MDA into law, introducing—at the time—a robust regulatory framework that included a three-tier risk-based classification system for devices; the Premarket Approval (PMA) pathway for high-risk devices, and the 510(k) premarket notification pathway for low- and moderate-risk devices. The MDA also established the Investigational Device Exemption (IDE) for clinical trials, including requirements for registration, listing, adverse event reporting, compliance with good manufacturing practices, and the authority for the FDA to recall devices found to be unsafe.

Arguably most significantly, the MDA included an express preemption clause under section 360k(a). This provision prohibits states from enacting or enforcing requirements “different from, or in addition to” federal requirements relating to a device's safety or efficacy. While the clause does not explicitly preempt state tort law (ie, products liability or medical malpractice claims), it specifically preempted California's medical device regulatory law that required premarket clearance through the state.^59^ The MDA clearly reallocated primary regulatory authority to the federal government, thus minimizing the risk of conflicting product regulation schemes, leaving oversight of use in real-world medical practice to states.

By 1976, the need for federal preemption was widely recognized. In the absence of federal regulation, states had attempted to fill the void, but their efforts were uneven and resulted in varying standards of safety and efficacy for medical devices, creating confusion for physicians and potentially exposing patients to unsafe devices. The preemption clause was designed to bring order to this chaos by ensuring that medical device product regulation would be governed by a single national standard administered by the FDA, while medical practice and device use would remain locally regulated by states.

While, at the time, the MDA balanced the tradeoffs of state regulation with unified federal product oversight, creating a national floor for safety and efficacy where one was lacking, 50 years later the device regulatory framework is due for updating. As the technological age progressed, analysts began denoting predicate device drift,^60^ increasing use of clearance over approval, and a greater need to modernize the evidentiary standard for market entry.^61^ While the initial tradeoffs the MDA brought were instrumental, a different assessment can be given half a century later, particularly in the AI era, highlighting the need for improvement in federal product market regulatory frameworks.

## Challenges with the current regulatory structure

The complexity of existing regulatory frameworks for many AI applications in healthcare, coupled with multiple regulatory participants from both state and federal levels, exposes patients to products of varying safety and efficacy across geographic boundaries due to a lack of a minimum regulatory floor and increases the difficulty and regulatory navigation costs for innovators. Many AI tools fall into categorical gray zones: they are not strictly “medical devices,” but they have clinical or operational effects (eg, triage support and resource allocation tools). Moreover, statutory and regulatory carve-outs (eg, wellness, administrative tools) leave entire classes of AI arguably unregulated by the FDA.^62^ Even for tools within FDA purview, their use in complex care settings frequently lies beyond FDA authority, as it would require ongoing monitoring and/or surveillance of human use of the AI system, raising privacy and security concerns in addition to practical operational issues. Moreover, even as hospitals begin to use AI, some of them, especially small or rural facilities,^63^ may lack the necessary expertise, data, and infrastructure to evaluate, monitor, and audit deployed AI tools. This can lead to a system in which harms (real or potential) are hard to capture, compensate, and correct.

In the absence of clearly delineated safe harbors, clear post-market surveillance frameworks, and appropriately scoped premarket safety testing, risk management, and remuneration for patient harms has been delegated into private contracts (ie, licensing and indemnification agreements), litigation, and patchwork state laws—none of which directly promote clear rules of the road for safety or efficacy of marketed products and simultaneously impose significant burdens on consumers seeking rectification of and compensation for harms. Private contracts with limited indemnification provisions sharply skew the allocation of legal risk to the human practitioner or hospital system using the AI product, increasing costs for the delivery system. Litigation is a steep burden for injured patients in light of the “black box” problem that obfuscates the AI algorithm's potential contribution to the injury,^64^ resulting in the patient's inability to be recompensated for an injury. The black box problem also increases the risk that AI algorithms trained on nonrepresentative data will result in conclusions that should not be applied to the patient at hand or otherwise replicate or magnify health disparities, further burdening individual hospital systems and practitioners with an unclearly scoped performance requirement for monitoring and local oversight. Finally, these processes do not facilitate either local actors, such as health systems, or centralized actors, such as a federal oversight agency like the FDA, to easily and systematically identify causes of errors and make improvements.

Some states have implemented increased oversight of “high risk AI”—with no carve out for health care applications nor medical devices—which may lead to inconsistent rules and compliance complexity. For example, a new product that fits the FDA's definition of a “wellness tool” and thus is exempt from premarket FDA approval would still have to meet state regulatory requirements in each state (such as Colorado and California) that has an AI-regulatory law on the books. Large and small developers alike may prioritize states with lax rules or avoid states with more restrictive oversight, leading to patients in certain states experiencing both differential access and differential product safety profiles.

While there is a valid federalism argument that “states should be the laboratories of democracy”^65^ and therefore should be left to decide these laws, medical devices fall outside the ambit of this argument. States should be the primary regulator for issues with locoregional differences in values (eg, building zoning regulations) or resource prevalence (eg, establishing a yearly hunting season for wildlife). For domains where consistency across state lines is paramount for national unity, however, federal law should likely prevail: for example, letting each state decide which side of the road drivers should use could lead to significant highway safety issues. Similarly, it is the role of the federal government to ensure pragmatic minimal technical floors to promote consumer protection in automobile technology or autopilot functions in commercial jetliners. Since medical devices ostensibly have the same safety and efficacy profile across state lines, national product standards can be used to create a floor for product safety, while the use of AI tools in clinical practice can and should be left up to the states.

## Future directions

State product regulation of AI, as currently unfolding, would encompass health care applications of AI that would be more effectively regulated at the federal level. Firstly, policymakers should build on pragmatic regulatory safe harbors akin to those executed in the 21st Century Cures Act. Next, policymakers could create a new, voluntary alternative fit-for-purpose FDA-based pathways for both integrated devices (devices that tightly integrate software components to drive the function of traditional medical devices and develop augmented or new capabilities)^66^ and pure autonomous software. Integrated devices may be best reviewed through a 2-stage approach, with the first stage requiring individual component review (ie, compliance with technical standards, modeling, etc.) and the second stage serving as a comprehensive review of the integrated device's performance. In contrast, pure autonomous software products may be best overseen through a total product lifecycle approach, with post-market surveillance a responsibility of the software developer in partnership with hospitals and clinicians as appropriate. So as not to disrupt existing analog device regulation, the FDA should maintain the current 510(k) and PMA review pathways as part of its risk-based regulatory framework, including the predetermined change control framework.^67,68^ To prepare for a world of software and AI in medicine, the FDA will also need to hire additional software engineering and AI talent, a future action assisted by existing Title 21 pay authority granted through the 21st Century Cures Act.^69,70^

Key principles in this approach would be execution based on the FDA's documented least burdensome principles,^71^ thoughtful safe harbors to promote incremental innovation, balanced with a requirement for pragmatic product oversight to ensure safety and efficacy of revolutionary innovation. A federal regulatory product scheme should include a statutory preemption provision, thus ensuring a minimum consumer protection floor for safety and efficacy while simultaneously creating clearer standards for market entry. Unlike the blanket moratorium proposed and rejected in the OBBB, a preemption provision should be limited to AI applications used in health care and be paired with improvements to FDA product oversight.

Critically, a unified, pragmatic federal product oversight framework in conjunction with state preemption would promote uniformity in product regulation, facilitating innovation and technological advancement through small businesses and ground-up product development from bedside clinicians in partnership with software developers. State-by-state fragmentation of regulatory requirements would disproportionately impact these grassroots innovators relative to big business. Federal product oversight would also establish clear boundaries for state action, respecting the autonomy and authority of states to regulate use in medical practice as they see fit.

Other jurisdictions have created frameworks for oversight of AI as a medical device. While European consumer digital privacy regulations have been criticized as overly obtrusive,^72^ the European Medicines Agency (EMA) has promoted the use of AI in medical product development by requiring automated, digital endpoint assessment,^73^ highlighting a pragmatic push toward automation in clinical trials.^74^ For software as a medical device validation, the EMA has focused on risk-based approaches, validation planning, and lifecycle management.^75,76^ Executed differently, these regulatory principles could support a pragmatic American approach to regulatory frameworks overseeing AI when coupled with thoughtful safe harbors and least burdensome principles.

## Conclusions

The current regulatory landscape for AI in health care is fragmented in a way that lacks the necessary coherence to ensure patient safety, promote innovation, and ensure American technological global leadership. The absence of a unified, flexible federal framework with sensible safe harbors is only being exacerbated by the growing patchwork of state laws. This creates uncertainty for innovators in terms of what premarket testing requirements and research and development costs will be required, and in some cases, leaves patient safety risks unmeasured or inadequately addressed. While state regulations aim at consumer protection, they introduce duplicative or additional requirements from state to state. To address these challenges, an express federal preemption provision should be paired with flexible, fit-for-purpose FDA-based review pathways with pragmatic regulatory safe harbors. This will help establish clear product standards and guidelines, require safeguards in all states rather than the current patchwork, and give clarity for innovators, medical practitioners, and patients.

## Disclaimers

Dr. Bucshon previously served as the Vice Chair of the Energy and Commerce; Health Subcommittee. Dr. Miller previously served as a Medical Officer at the US Food and Drug Administration. Dr. Yagi previously served as a legal intern in the Office of Policy and Planning at the FDA. Views are the authors' own and not necessarily those of their employers or affiliations.

## Supplementary Material

qxag046_Supplementary_Data
